# Dependence of detectability of microcalcification clusters on quality of mammography images

**DOI:** 10.1186/bcr2975

**Published:** 2011-11-04

**Authors:** LM Warren, A Mackenzie, J Cooke, R Given-Wilson, M Wallis, D Chakraborty, DR Dance, KC Young

**Affiliations:** 1 National Co-ordinating Centre for the Physics of Mammography, Guildford, UK; 2 Jarvis Breast Screening and Diagnostic Centre, Guildford, UK; 3 St George's Healthcare NHS Trust, London, UK; 4 Cambridge University Hospitals NHS Foundation Trust, Cambridge, UK; 5 University of Pittsburgh, PA, USA; 6 University of Surrey, Guildford, UK

## Introduction

This work compares the detection of microcalcification clusters (MC) in digital mammography images with different image quality levels.

## Methods

A total of 162 normal breast images were acquired on an a-Se DR system. MC clusters extracted from magnified images of sliced mastectomies were electronically inserted into half of the images. The majority of clusters used were subtle. All images were adjusted mathematically using a validated method to simulate the appearance of images from a CR imaging system at the same dose and on both systems at half this dose. Seven experienced observers marked the location of suspicious regions, assigning a five-point score for confidence that the suspicious region was a cluster. The data were analysed using the area under the alternative free-response receiver operating characteristic (AFROC) and the area under the receiver operating characteristic (ROC) as figures of merit.

## Results

There was a significant reduction in detection using CR compared with DR; the AFROC area decreased from 0.83 to 0.63 and the ROC area decreased from 0.91 to 0.79 (*P *< 0.0001). A significant reduction in detection was also evident at half the original dose for both DR and CR.

## Conclusion

The detection of subtle clusters was reduced significantly with CR compared with DR and it is possible that CR will miss cancers manifesting as microcalcification that would be found by DR. Calcification detection was sensitive to the dose used, which should be reflected in image quality standards to ensure adequate image quality is achieved even at the cost of a higher dose.

